# Evolution of a reassortant North American gull influenza virus lineage: drift, shift and stability

**DOI:** 10.1186/1743-422X-10-179

**Published:** 2013-06-06

**Authors:** Jeffrey S Hall, Joshua L TeSlaa, Sean W Nashold, Rebecca A Halpin, Timothy Stockwell, David E Wentworth, Vivien Dugan, Hon S Ip

**Affiliations:** 1 Current address: USGS National Wildlife Health Center, 6007 Schroeder Rd, Madison, WI, USA; 2 USGS National Wildlife Health Center, Madison, WI, USA; 3 J Craig Venter Institute, 9704 Medical Center Drive, Rockville, MD, USA

## Abstract

**Background:**

The role of gulls in the ecology of avian influenza (AI) is different than that of waterfowl. Different constellations of subtypes circulate within the two groups of birds and AI viruses isolated from North American gulls frequently possess reassortant genomes with genetic elements from both North America and Eurasian lineages. A 2008 isolate from a Newfoundland Great Black-backed Gull contained a mix of North American waterfowl, North American gull and Eurasian lineage genes.

**Methods:**

We isolated, sequenced and phylogenetically compared avian influenza viruses from 2009 Canadian wild birds.

**Results:**

We analyzed six 2009 virus isolates from Canada and found the same phylogenetic lineage had persisted over a larger geographic area, with an expanded host range that included dabbling and diving ducks as well as gulls. All of the 2009 virus isolates contained an internal protein coding set of genes of the same Eurasian lineage genes except PB1 that was from a North American lineage, and these genes continued to evolve by genetic drift. We show evidence that the 2008 Great Black-backed Gull virus was derived from this lineage with a reassortment of a North American PA gene into the more stable core set of internal protein coding genes that has circulated in avian populations for at least 2 years. From this core, the surface glycoprotein genes have switched several times creating H13N6, H13N2, and H16N3 subtypes. These gene segments were from North American lineages except for the H16 and N3 vRNAs.

**Conclusions:**

This process appears similar to genetic shifts seen with swine influenza where a stable “triple reassortant internal gene” core has circulated in swine populations with genetic shifts occurring with hemaggluttinin and neuraminidase proteins getting periodically switched. Thus gulls may serve as genetic mixing vessels for different lineages of avian influenza, similar to the role of swine with regards to human influenza. These findings illustrate the need for continued surveillance in gull and waterfowl populations, both on the Pacific and especially Atlantic coasts of North America, to document virus intercontinental movement and the role of gull species in the evolution and epidemiology of AI.

## Introduction

The principle reservoirs of avian influenza virus (AIV) are wild waterfowl (Anseriformes) and shorebirds and gulls (Charadriiformes) [1]. Most studies to date have focused primarily on ducks and geese during their autumn migration as well as shorebirds at Delaware Bay, USA in the spring. However evidence is accumulating that influenza ecology in gulls is different than in waterfowl and warrants increased research efforts.

Because AIV has a genome comprised of eight RNA segments (vRNAs), if a host cell becomes infected with two or more viruses, these vRNAs can reassort and the resulting progeny may contain hybrid genomes of the parental viruses. This process is called reassortment and can result in antigenic shifts that dramatically alter host range, pathology, and transmission of the virus.

Due to limited genetic interaction, avian influenza viruses have evolved into two phylogenetically distinct populations, a North American lineage and a Eurasian lineage [2,3]. To date, no completely Eurasian influenza virus has been isolated in North America, however there is increasing evidence that reassortment between the lineages occurs regularly. Reassortant viruses have been documented in Northern Europe and in North America, including from shorebirds and gulls at Delaware Bay [3]. Alaska and areas of Asia adjacent to the Bering Sea are sites of active interlineage reassortment [4-6]. This is not surprising given the proximity of Asia and North America in that region and that large numbers of birds cross between the continents during seasonal migrations.

Gull species can be wide ranging, often moving between continents, and congregate in large groups both on and off the breeding sites, increasing the likelihood of transmission of AIV between birds and also from the environment or food sources. Emerging evidence indicates that influenza virus ecology in gulls differs from that in waterfowl with different constellations of subtypes circulating within the two groups of birds [7,8]. AI viruses isolated from North American gulls also frequently contain reassortant genomes with genetic elements from both North America and Eurasian lineages [6,9].

In this study we document the continuing evolution of a gull lineage of avian influenza virus from the North Atlantic coast of Eastern Canada. We found evidence of a semi-stable core set of internal protein coding genes around which the surface glycoprotein genes have reassorted. We also found that the host range of this gull virus lineage has expanded into both diving and dabbling ducks and that the geographic range of this lineage is larger than previously thought. These findings highlight the continuing need to monitor influenza viral lineages in Canadian and US Atlantic regions, in both gulls and waterfowl, to document virus evolution and movement of viruses and viral genetic elements between North America and Europe.

## Methods

### Sample acquisition

2719 cloacal swab or combination oral/cloacal swab samples were collected from 28 wild bird species by wildlife professionals of the Canadian Cooperative Wildlife Health Centre in 2009. Birds were captured by a variety of methods at numerous locations on the Atlantic coast of Canada (provinces of Nunavut, Prince Edward Island, New Brunswick, Nova Scotia, Quebec). Swabbing utilized Dacron tipped applicators that were placed in cryovials containing viral transport media and stored in liquid nitrogen or at −80°C until shipping to the USGS National Wildlife Health Center where they were stored at −80°C until analyses. All procedures were approved by the appropriate Institutional Animal Care and Use Committees.

### Real time-reverse transcription polymerase chain reaction (RT-PCR) and virus isolation

Viral RNA was extracted using the MagMAX™-96 AI/ND Viral RNA Isolation Kit (Ambion, Austin, TX) following the manufacturer’s procedures. Real time RT-PCR was performed using the published procedures, primers, and probe of Spackman et al. [10] designed to detect the influenza A virus matrix gene. RT-PCR assays used reagents provided in the Qiagen OneStep® RT-PCR kit. Virus isolation was attempted on all swabs exhibiting positive Ct values from RT-PCR analysis. Virus isolation was performed in embryonating chicken egg culture according to the methods described by Woolcock [11].

### Sequencing of virus isolates and phylogenetic analysis

Virus isolates were sequenced at the J. Craig Venter Institute through the NIH/NIAID sponsored Influenza Genome Sequencing Center. Viral RNA was extracted from the isolates and amplified by multisegment RT-PCR [12]. The cDNA amplicons were barcoded for highly parallel sequencing using SISPA and randomly primed, as previously described [13]. Amplicons were sequenced on both the 454/Roche-FLX sequencing platform and Illumina HiSeq 2000 platforms and both data sets were used for gene segment assemblies. Related viruses were determined using BLAST searches of influenza virus sequences in GenBank. Sequences were aligned with representative Eurasian and North American viruses using Clustal W [14]. Phylogenetic and molecular evolutionary analyses were conducted using *MEGA* version 5.0 [15]. Maximum likelihood phylogenetic trees of the internal protein coding vRNAs were rooted using the sequences of A/duck/Novosibirsk/02/05 (H5N1).

## Results

From 2719 swab samples taken in 2009, we were able to isolate 55 avian influenza viruses from Canadian birds, primarily from waterfowl in the Maritime Provinces. Genomic sequencing of these isolates and phylogenetic comparisons of each full length RNA segment revealed that the majority of the viruses were related to North American duck viruses (not shown). However a group of 5 virus isolates (Table 1) consistently formed a distinct clade, across all viral gene segments. Two of these viruses were isolated from ring-billed gulls (*Larus delawarensis*) (A/ring-billed gull/Quebec/02622-1/2009(mixed); A/ring-billed gull/Quebec/02434-1/2009(H13N6)), one from another gull species, black-legged kittiwake (*Rissa tridactyla*) (A/black-legged kittiwake/Quebec/02838-1/2009(H13N6)), and one each from a mallard x American black duck (*Anas rubripes*) hybrid (A/mallard-black duck hybrid/New Brunswick/03736/2009(H13N6)), and hooded merganser (*Lophodytes cucullatus*) (A/hooded merganser/New Brunswick/03750/2009(H13N6)). Another virus isolate, (A/mallard/Quebec/02916-1/2009(H16N3)) also grouped with the other viruses across all of the internal protein coding RNA segments. The ring-billed gull isolate that was a mixed infection based on sequence analysis, was infected with H13N6 as the predominant virus subtype present and those sequences were used for all subsequent analysis.

**Table 1:** 2009 Epidemiological data on 6 gull lineage virus isolates obtained from wild bird samples

**Virus isolate**	**Location**	**Date**	**Age**^**1**^	**Sex**
A/RBGU^2^/Quebec/02622-1/2009 (mixed)*	Beauport, QC	7/20/2009	HY	Male
A/RBGU/Quebec/02434-1/2009 (H13N6)*	Saint-Joachim-de-Shefford, QC	7/9/2009	AHY	Female
A/HOME^3^/New Brunswick/03750/2009 (H13N6)	Rush Lake, NB	9/14/2009	HY	Female
A/BLKI^4^/Quebec/02838-1/2009 (H13N6)*	Cap-Bon-Ami, QC	8/11/2009	HY	Unknown
A/MALL^5^/Quebec/02916-1/2009 (H16N3)*	Gatineau, QC	8/18/2009	HY	Male
A/MBDH^6^/New Brunswick/03736/2009 (H13N6)	Rush Lake, NB	9/14/2009	HY	Male

^1^Age expressed as HY (Hatch Year) or AHY (After Hatch Year).

^2^Ring-billed gull (*Larus delawarensis*); ^3^Hooded merganser (*Lophodytes cucullatus*); ^4^Black-legged kittiwake (*Rissa tridactyla*); ^5^Mallard (*Anas platyrhynchos*); ^6^Mallard x American black duck (*Anas rubripes*) hybrid.

*These influenza isolates obtained from dead bird submissions.

**Figure 1:**
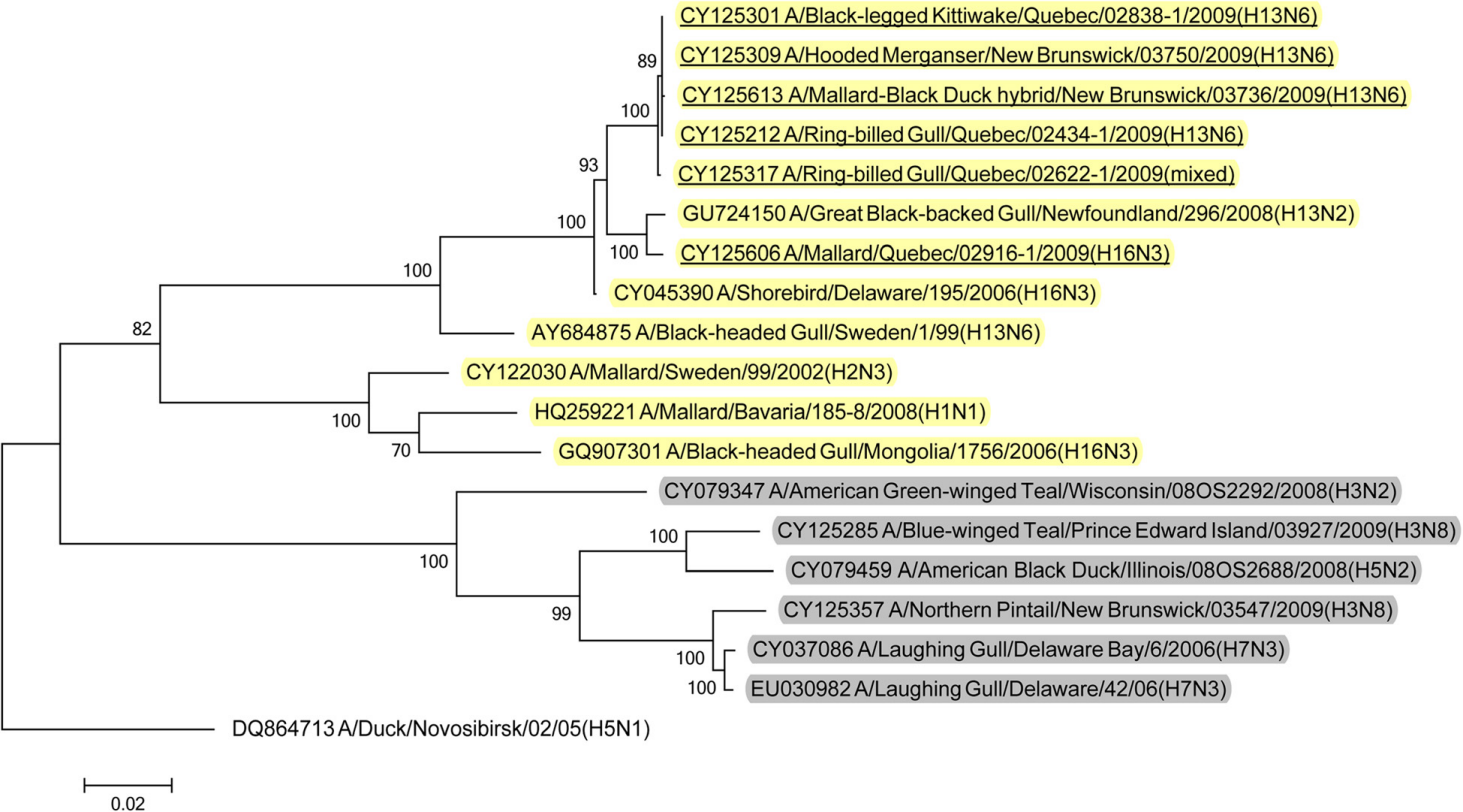
**Maximum likelihood phylogenetic tree of PB2 gene of Canadian isolates and representative Eurasian (highlighted in yellow) and North American (gray) lineage influenza viruses.** 2009 Canadian virus isolates are underlined.

**Figure 2:**
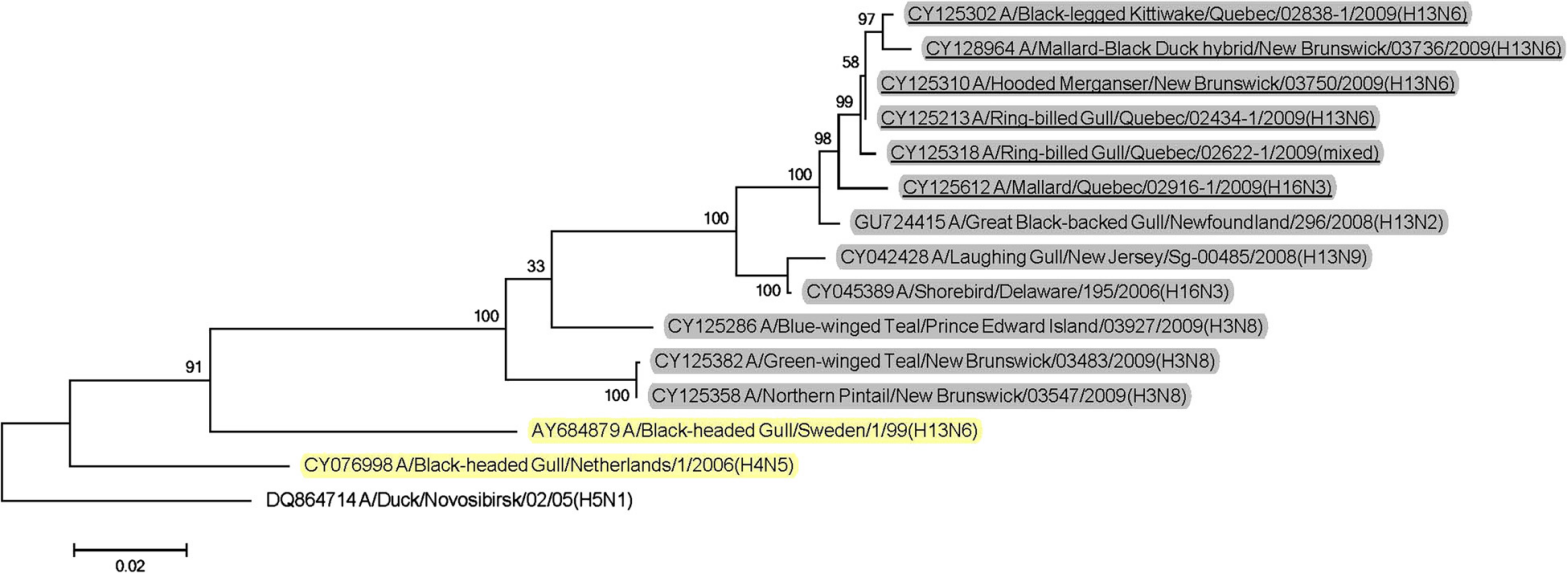
**Maximum likelihood phylogenetic tree of PB1 gene of Canadian isolates and representative Eurasian (highlighted in yellow) and North American (gray) lineage influenza viruses.** 2009 Canadian virus isolates are underlined.

**Figure 3:**
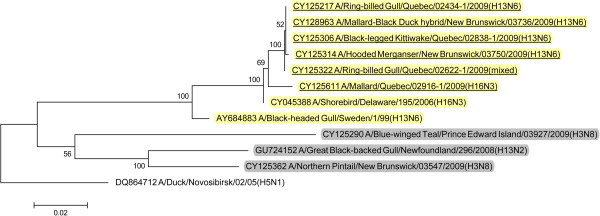
**Maximum likelihood phylogenetic tree of PA gene of Canadian isolates and representative Eurasian (highlighted in yellow) and North American (gray) lineage influenza viruses.** 2009 Canadian virus isolates are underlined.

**Figure 4:**
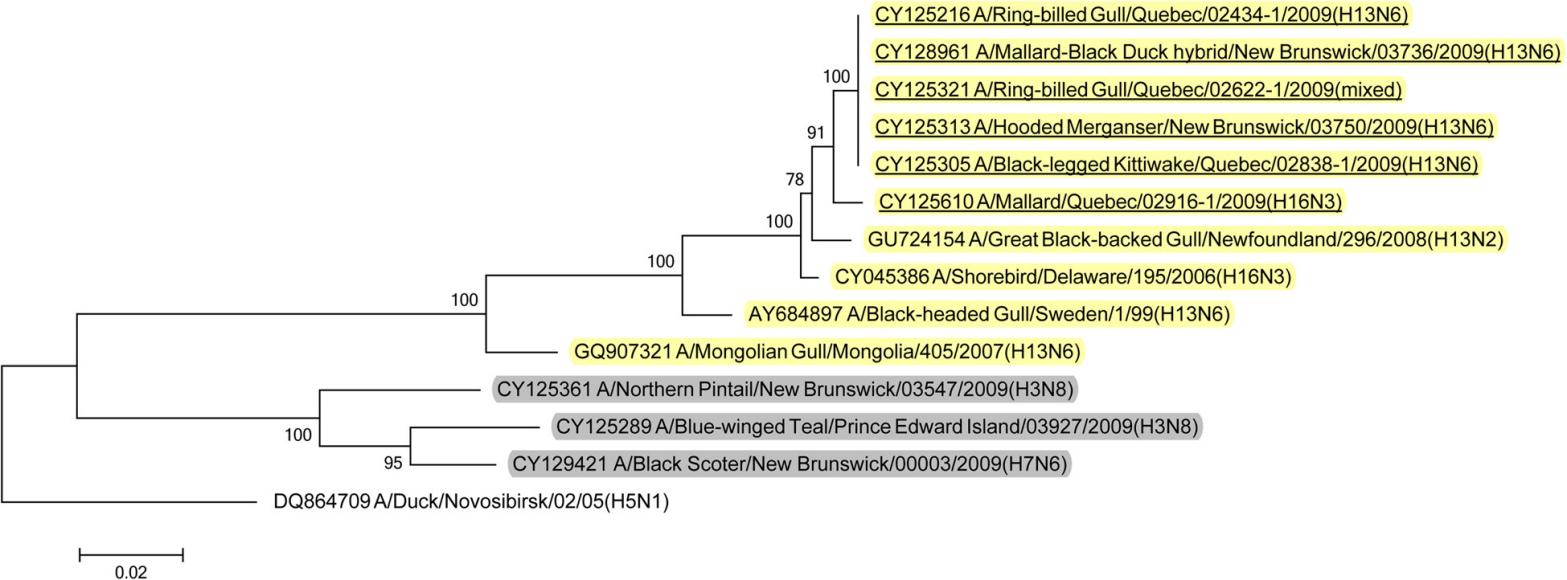
**Maximum likelihood phylogenetic tree of NP gene of Canadian isolates and representative Eurasian (highlighted in yellow) and North American (gray) lineage influenza viruses.** 2009 Canadian virus isolates are underlined.

**Figure 5:**
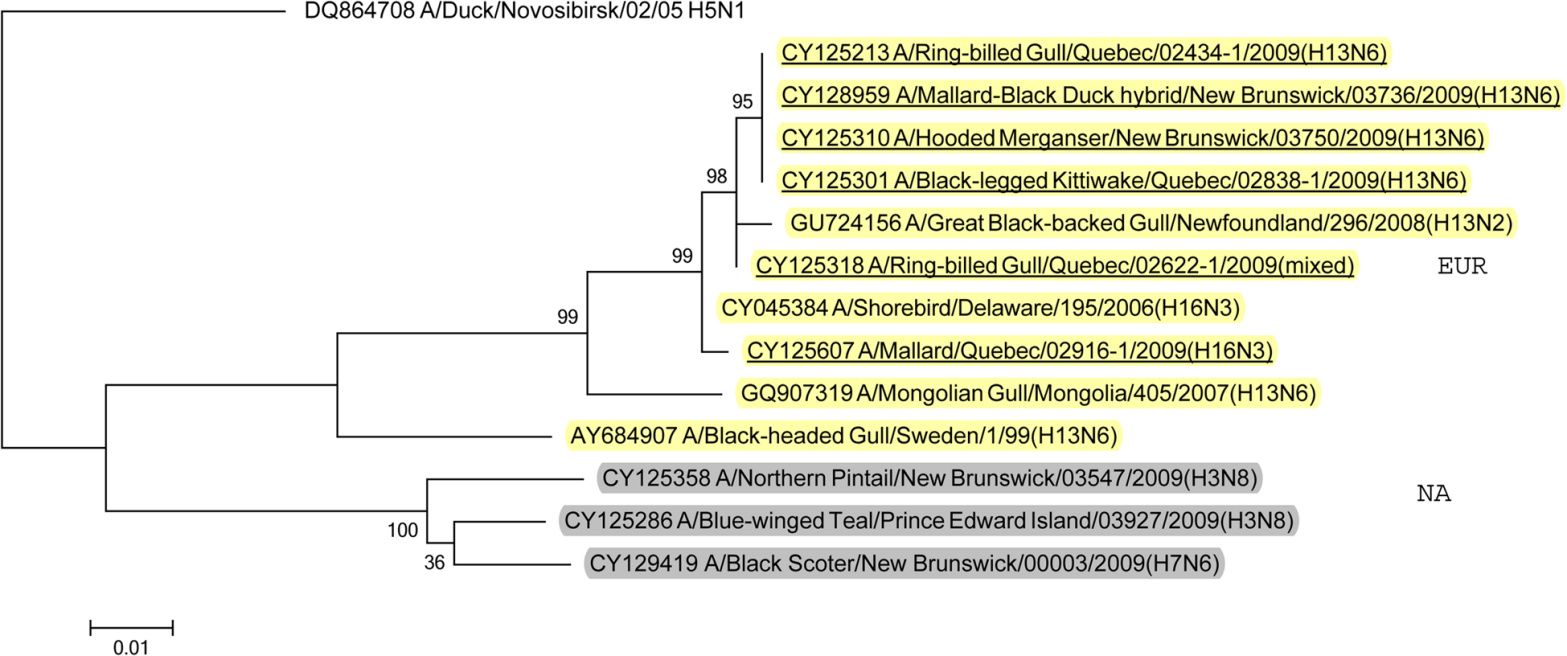
**Maximum likelihood phylogenetic tree of Matrix gene of Canadian isolates and representative Eurasian (highlighted in yellow) and North American (gray) lineage influenza viruses.** 2009 Canadian virus isolates are underlined.

**Figure 6:**
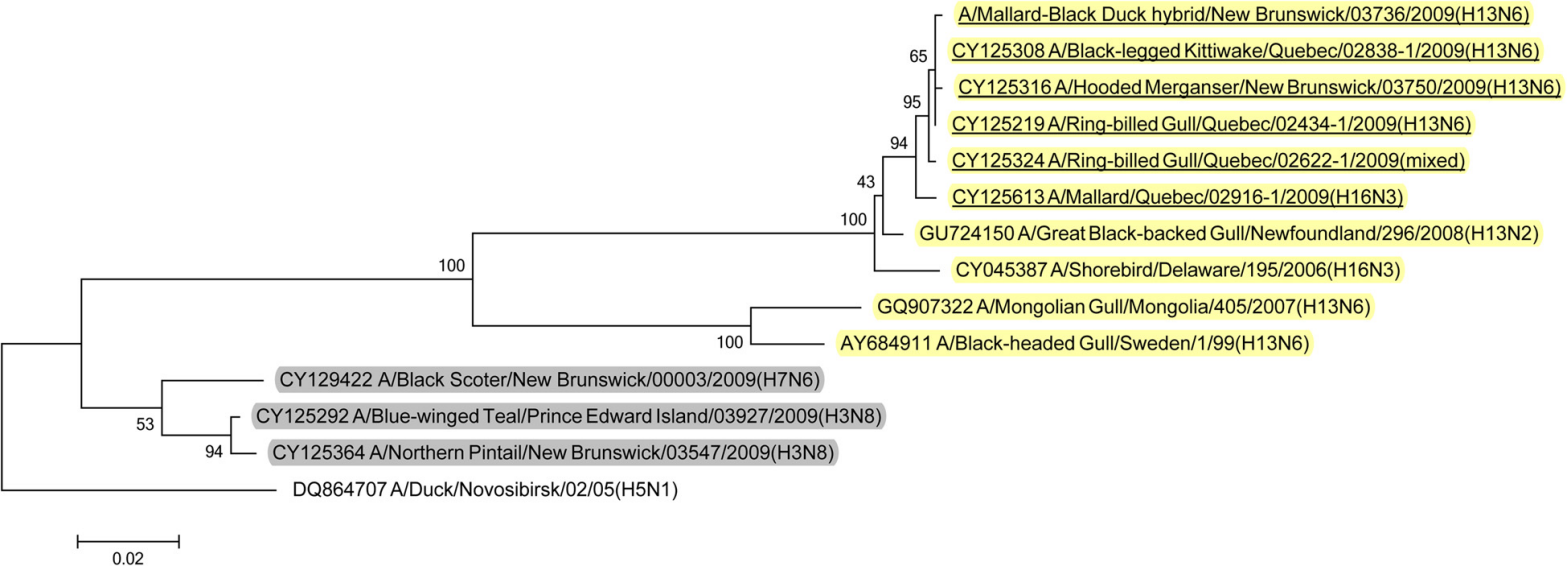
**Maximum likelihood phylogenetic tree of NS gene of Canadian isolates and representative Eurasian (highlighted in yellow) and North American (gray) lineage influenza viruses.** 2009 Canadian virus isolates are underlined.

BLAST queries of sequence databases indicated that all but the PA gene of these six viruses’ internal protein coding genes were closely related to a recently described H13N2 influenza virus isolated from a 2008 great black-backed gull (GBBG) in Newfoundland, Canada (A/great black-backed gull/Newfoundland/296/2008 (H13N2)). This virus was previously shown to have a mosaic genome containing genes from a Eurasian lineage (PB2, NP, M, NS), North American gull lineage (PB1, HA) and North American waterfowl lineage (PA, NA) [9]. All six of the 2009 viruses contained PA genes of Eurasian lineage, and thus all of their internal protein genes (M, NP, NS, PA, PB2) were of Eurasian origin with the exception of PB1 that belonged to the North American lineage. Maximum likelihood phylogenetic trees illustrating the relationships of the six 2009 viruses’ internal protein genes to the GBBG 2008 virus and representative Eurasian and sympatric North American viruses are shown in Figures 1, 2, 3, 4, 5, 6; Table 2. Phylograms computed using other methods (maximum parsimony, neighbor joining) showed similar topologies (not shown).

**Table 2:** Genomic structure of reassortant avian influenza viruses isolated from Canadian North Atlantic birds

	**Gene segment**							
**Isolate**	**PB2**	**PB1**	**PA**	**HA**	**NP**	**NA**	**Matrix**	**NS**
A/GBBG^1^/Newfoundland/296/2008 (H13N2)	Eur^7^	NAG	NAW	NAG	Eur	NAW	Eur	Eur
A/RBGU^2^/Quebec/02622-1/2009 (mixed)	Eur	NAG	Eur	NAG	Eur	NAW	Eur	Eur
A/RBGU/Quebec/02434-1/2009 (H13N6)	Eur	NAG	Eur	NAG	Eur	NAW	Eur	Eur
A/HOME^3^/New Brunswick/03750/2009 (H13N6)	Eur	NAG	Eur	NAG	Eur	NAW	Eur	Eur
A/BLKI^4^/Quebec/02838-1/2009 (H13N6)	Eur	NAG	Eur	NAG	Eur	NAW	Eur	Eur
A/MALL^5^/Quebec/02916-1/2009 (H16N3)	Eur	NAG	Eur	Eur	Eur	Eur	Eur	Eur
A/MBDH^6^/New Brunswick/03736/2009 (H13N6)	Eur	NAG	Eur	NAG	Eur	NAW	Eur	Eur

^1^Great black-backed gull (*Larus marinus*): lineage data from Wille et al. 2010.

^2^Ring-billed gull (*Larus delawarensis*); ^3^Hooded merganser (*Lophodytes cucullatus*); ^4^Black-legged kittiwake (*Rissa tridactyla*); ^5^Mallard (*Anas platyrhynchos*); ^6^Mallard x American black duck (*Anas rubripes*) hybrid.

^7^Gene segment lineages characterized as Eurasian (Eur); North American Waterfowl (NAW), or North American Gull (NAG).

**Figure 7:**
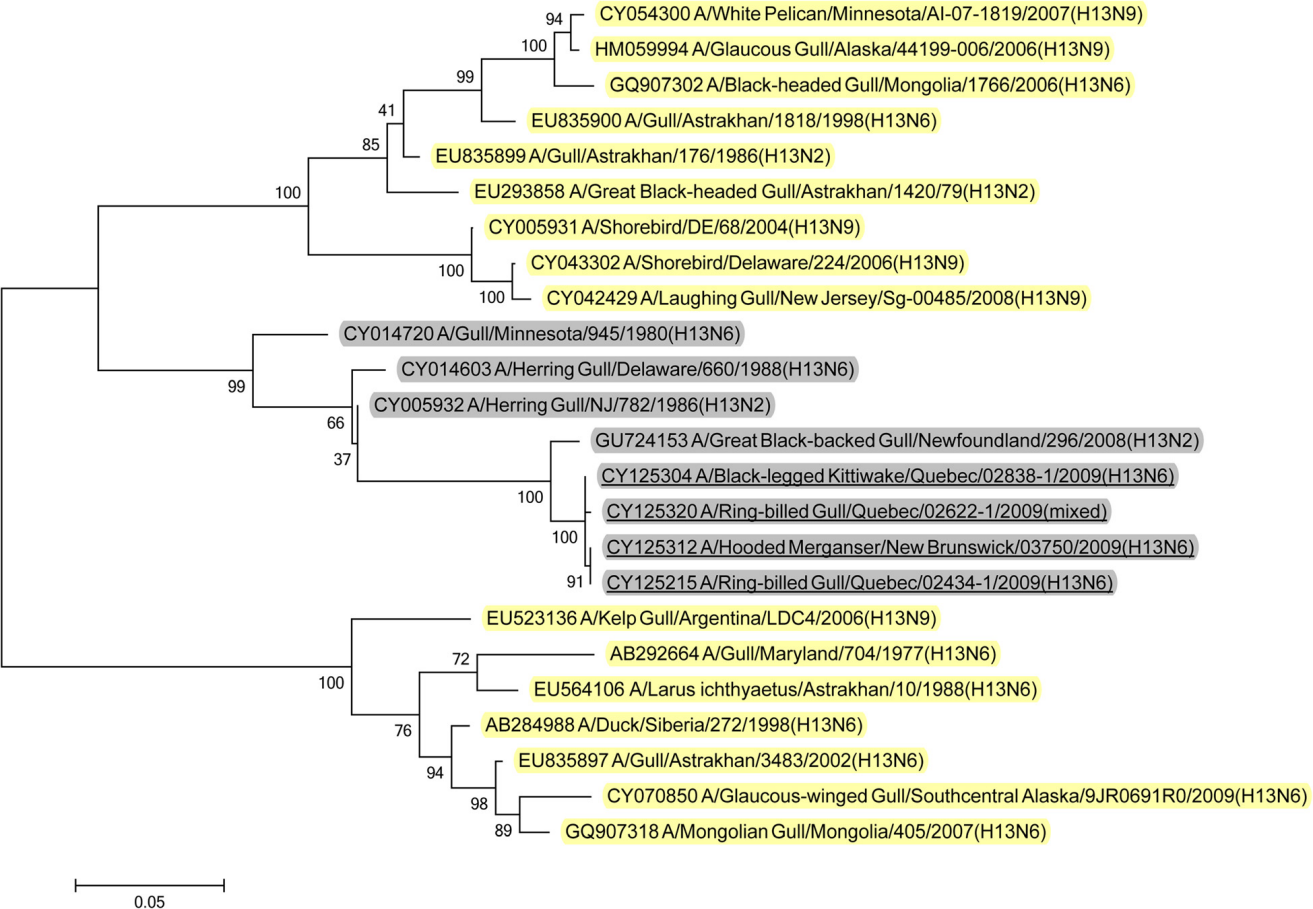
**Maximum likelihood phylogenetic tree of H13 genes of Canadian isolates and representative Eurasian (highlighted in yellow) and North American (gray) lineage influenza viruses.** 2009 Canadian virus isolates are underlined.

**Figure 8:**
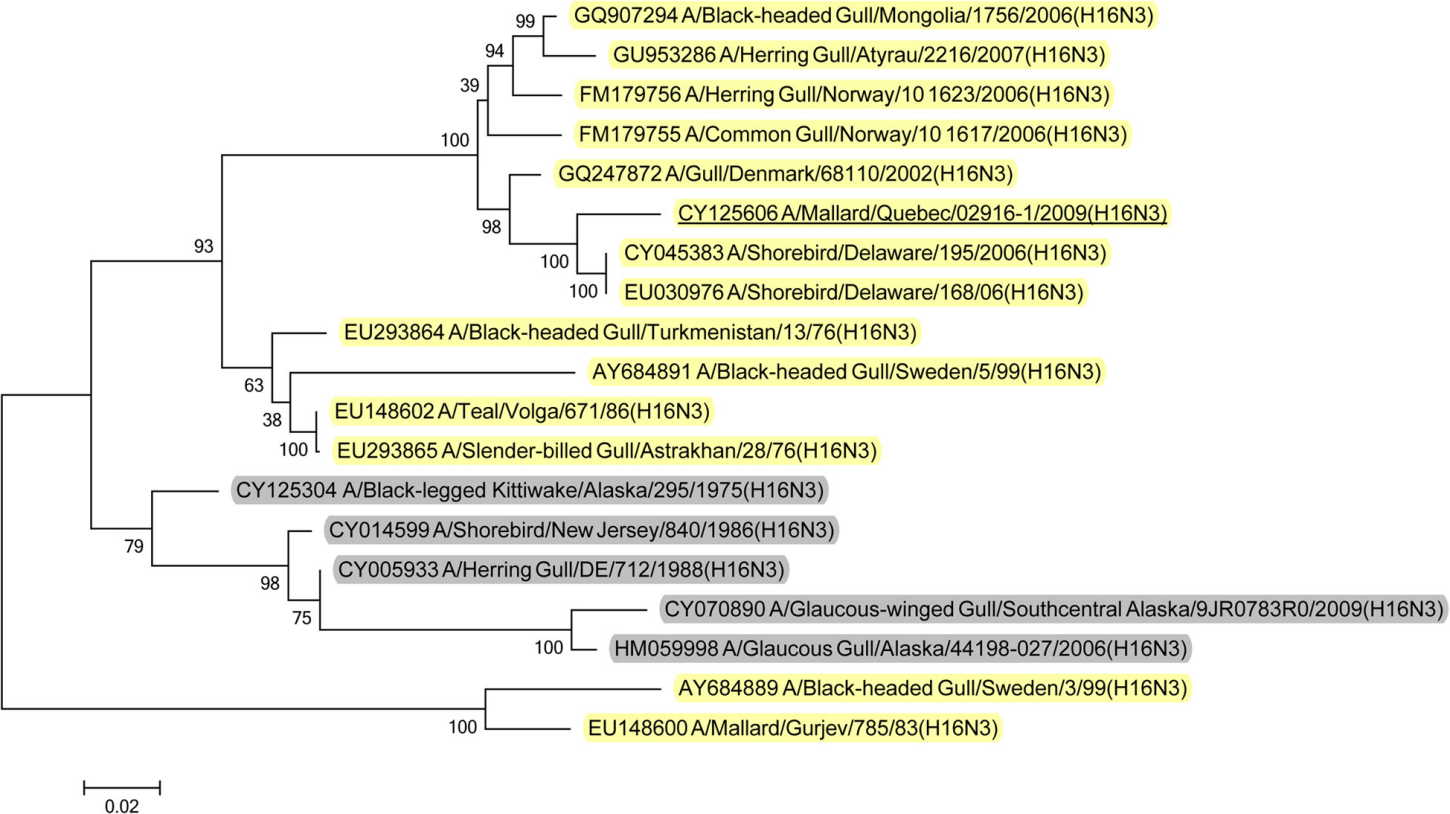
**Maximum likelihood phylogenetic tree of H16 genes of Canadian isolate and representative Eurasian (highlighted in yellow) and North American (gray) lineage influenza viruses.** 2009 Canadian virus isolates are underlined.

**Figure 9:**
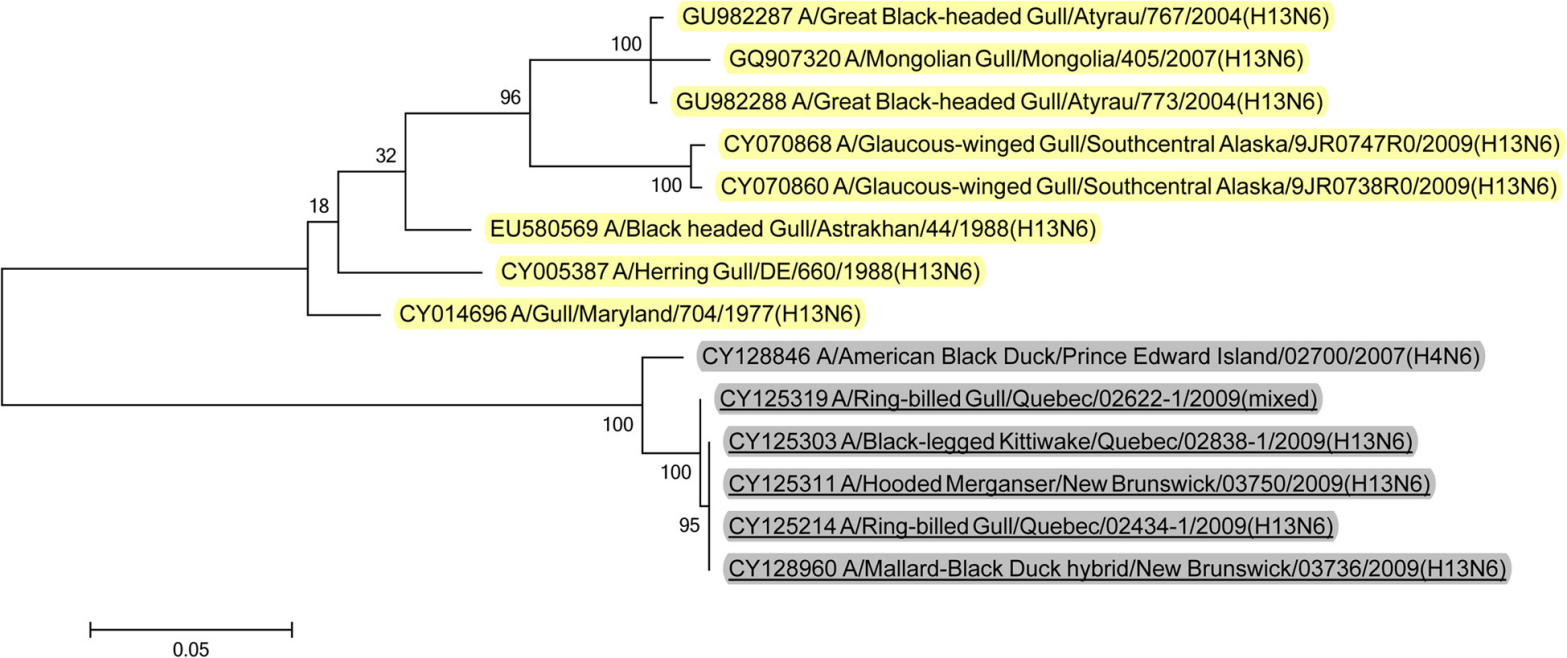
**Maximum likelihood phylogenetic tree of N6 genes of Canadian isolates and representative Eurasian (highlighted in yellow) and North American (gray) lineage influenza viruses.** 2009 Canadian virus isolates are underlined.

**Figure 10:**
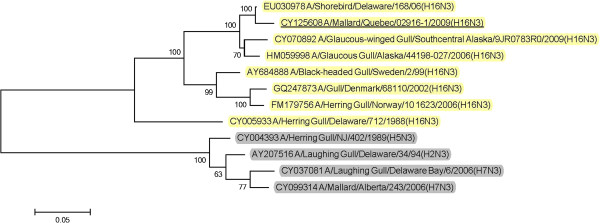
**Maximum likelihood phylogenetic tree of N3 genes of Canadian isolate and representative Eurasian (highlighted in yellow) and North American (gray) lineage influenza viruses.** 2009 Canadian virus isolates are underlined.

Analysis of the surface glycoprotein genes showed that the five Canadian H13N6 viruses from 2009 possessed hemagglutinin (HA) genes that were in a North American gull lineage that also contained the 2008 GBBG virus (Figure 7). However, the HA gene from the 2009 mallard H16N3 virus was from a Eurasian background (Figure 8). The N6 neuraminidase (NA) genes were from a North American lineage (Figure 9) and the mallard N3 gene was from a Eurasian lineage (Figure 10).

## Discussion

In this study we documented the continuing evolution of a reassortant gull influenza lineage in Eastern Canada. In 2008 an influenza virus was isolated from a great black-backed gull in Newfoundland and was found to contain genes from Eurasian, North American gull, and North American waterfowl lineages [9]. In 2009 we isolated viruses from Canadian gulls, a diving duck, and two dabbling ducks that had five of their internal protein coding genes phylogenetically closely related to the 2008 GBBG virus. The other internal protein gene, PA, was Eurasian lineage in all of the 2009 viruses but was North American lineage in the GBBG virus. Interestingly, all of these virus’ internal gene phylogenies except the GBBG PA gene, show a common ancestor, a virus isolated from a shorebird sampled at Delaware Bay, USA in 2006 (A/shorebird/Delaware/195/2006 (H16N3)). Since we can’t recover the complete evolutionary history of these viruses, based on the single virus isolate in 2008 and these 6 from 2009, and the 2006 shorebird virus, it seems likely that the ancestral source of this virus lineage was Eurasian H16N3, and a reassortant event occurred where a North American PB1 gene was switched into the Eurasian genetic backbone. Thus, over at least four years, a seemingly stable influenza internal protein coding gene core consisting of five Eurasian (PB2, PA, M, NS, NP) and one North American (PB1) gene lineages has formed in gull and duck populations in the Canadian Atlantic region. With the exception of the reassortment event in the GBBG virus where the PA gene was switched to a North American lineage, only genetic drift occurred within these gene lineages over the four years of virus isolates that have been examined.

Around this stable core set of genes, however, the viral surface glycoproteins have switched several times with three different subtypes circulating, H13N2, H13N6, and H16N3. These genetic shifts have apparently expanded the host range of this gull lineage to include both diving and dabbling waterfowl. These findings may be similar to the situation with swine influenza where a stable “triple reassortant internal gene” (TRIG) core has been the predominant influenza genotype circulating since 1999 with the surface proteins getting switched more frequently [16]. It will be interesting to monitor these virus populations in subsequent years to see if the stability of this lineage is maintained and expands both in terms of prevalence, geography and host range.

Interestingly, four of the six 2009 viruses were isolated from dead birds submitted for diagnostics to Canadian Wildlife Disease specialists (A/ring-billed gull/Quebec/02622-1/2009(mixed); A/ring-billed gull/Quebec/02434-1/2009(H13N6); A/black-legged kittiwake/Quebec/02838-1/2009(H13N6); A/mallard/Quebec/02916-1/2009(H16N3)). It is not known whether infection with these low pathogenic AI viruses was a direct cause of the mortality or if they were contributing factors with another pathogen infection or disease. Infection with LPAIV typically causes few if any disease signs and the fact that similar viruses were isolated from an apparently healthy hooded merganser and a mallard X black duck hybrid suggests that these viruses don’t cause morbidity or mortality in waterfowl by themselves in these hosts.

To date no wholly Eurasian influenza viruses have been found in North America. The H16N3 virus isolates (A/mallard/Quebec/02916-1/2009(H16N3); A/shorebird/Delaware/195/2006 H16N3)) are the closest with all genes except PB1 coming from Eurasian lineages. Clearly, based on the frequency of reassortant viruses found in North American gulls, these birds come into contact with Eurasian viruses or with birds transmitting those viruses. Gulls can have very large ranges and the potential exists for these birds to transport influenza viruses, including highly pathogenic avian influenza virus H5N1, intercontinentally between the Old and New Worlds. Continued monitoring of virus populations in gull species is important for understanding influenza virus evolution, potential generation of new subtypes, and risk analyses of pathogenic strains introduction into new geographic regions.

## Conclusions

We have documented the existence of a reassortant influenza virus lineage in 2009 Canadian Atlantic wild birds. This lineage contains Eurasian and North American gene segments and is likely an ancestor of a previously described virus isolated from a great black-backed gull in 2008. This ancestral lineage contains a stable core set of internal protein coding gene segments about which the surface glycoprotein genes, hemagglutinin and neuraminidase, have been switched several times. These findings support the need for continued monitoring of wild bird populations in the North Atlantic for avian influenza virus movement, reassortment, and evolution.

## Competing interests

The authors declare that they have no competing interests.

## Authors’ contributions

JSH designed, planned, and coordinated the study, analyzed the data and wrote the manuscript. JLT contributed to the planning, laboratory analyses, data analyses and manuscript preparation. SWN contributed to the planning, laboratory analyses, data analyses and manuscript preparation. RAH, TS, VD, DEW sequenced the virus isolates and contributed to the analyses. HSI contributed to the study planning, data analyses and manuscript preparation. All authors have read and approved the final manuscript.
